# Association Between Indices of Body Composition and Metabolically Unhealthy Phenotype in China: A Cross-Sectional Study

**DOI:** 10.3389/fendo.2022.891327

**Published:** 2022-05-09

**Authors:** Fang Lv, Xiaoling Cai, Yufeng Li, Zuodi Fu, Xiuying Zhang, Xianghai Zhou, Xueyao Han, Linong Ji

**Affiliations:** ^1^ Department of Endocrinology and Metabolism, Peking University People’s Hospital, Beijing, China; ^2^ Department of Endocrinology, Beijing Friendship Hospital, Beijing, China

**Keywords:** visceral adipose, subcutaneous adipose, skeletal muscle, metabolically healthy, obesity

## Abstract

**Introduction:**

Body composition is closely related to metabolic health status. Visceral adipose tissue (VAT) dysfunction contributes to metabolic syndrome. However, results regarding subcutaneous adipose tissue (SAT) and skeletal muscle are controversial. We aimed to determine the association of indices of body composition with abnormal metabolic phenotype in China.

**Methods:**

A total of 3, 954 subjects (age 50.2 ± 11.7 years) with body mass index (BMI) more than 18.5 kg/m^2^ from Pinggu Metabolic Disease Study were analyzed. Quantitative computed tomography (QCT) was performed to measure total adipose tissue (TAT), VAT, SAT area, and lumbar skeletal muscle area (SMA). Participants were divided into six groups on the basis of BMI category (normal weight/overweight/obesity) and metabolic status (healthy/unhealthy), as defined by the presence or absence of components of the metabolic syndrome by Chinese Diabetes Society criteria.

**Results:**

63.4%, 39.5%, and 23.3% participants were classified as metabolically healthy phenotype in individuals with normal weight, overweight and obese, respectively. Individuals in the highest TAT, VAT, and VAT/TAT ratio category had higher risk of being metabolically unhealthy than individuals in the lowest group (all *p*<0.01). While, risk for metabolically unhealthy was reduced significantly in the highest SMA/TAT ratio category when compared with the lowest category in individuals with normal wight and overweight (both *p*<0.05). Risk for metabolically unhealthy was reduced significantly in the highest SAT category when compared with the lowest category (OR=0.555, 95%CI: 0.360-0.856, *p*=0.008) in individuals with obese after adjustment for age, sex and BMI. However, skeletal muscle index (SMI) showed no significant association with the metabolically healthy status in different BMI categories (*p*>0.05). The VAT and VAT/TAT ratio were better diagnostic values of indicators to differentiate metabolically unhealthy subjects from controls compared with other indicators, such as TAT, SAT, SMI, SMA/TAT ratio.

**Conclusions:**

Higher visceral adipose tissue was closely associated with metabolically unhealthy phenotype in Chinese adults. Subcutaneous adipose tissue might be a protective factor for metabolic health status only in obese individuals.

## Introduction

Obesity is a serious health problem, which alters the state of metabolism, leading to dyslipidemia, insulin resistance, and inflammation, and is therefore an important risk factor for cardiovascular diseases (CVD) and type 2 diabetes (T2D) (1). However, individuals in the same body mass index (BMI) category can have substantial heterogeneity of metabolic features. Individuals of “metabolically healthy obesity” (MHO) phenotype may not be at an increased risk for cardiovascular complications, and have a favorable lipid and glucose profiles (2, 3). In contrast, not all non-obese individuals present with a healthy metabolic profile, and were described to have “metabolically unhealthy non-obesity (MUNO)” (3, 4). If people with different healthy status could be identified, this could mean great benefits both for the individual and the health care system.

Body composition is closely related to metabolic health status. Previous studies indicated that MHO individuals were characterized by lower visceral fat mass, less fat accumulation in liver and skeletal muscle as compared to metabolically unhealthy obese (MUO) individuals (5–7), emphasizing the role of adipose tissue function in metabolic health. The effects of subcutaneous adipose tissue on metabolic health status are complex. Some studies supported beneficial effects of subcutaneous adipose tissue on metabolic health status while others not (8–13). Moreover, abdominal subcutaneous adipose tissue mass and gluteofemoral fat mass might have different effects on metabolic health status. German Tübingen diabetes family study showed that more metabolically unhealthy patients had a high percentage of subcutaneous abdominal fat mass, and a low percentage of subcutaneous gluteofemoral and leg fat mass, than did metabolically healthy patients (12, 13). However, the Dallas Heart Study did not show that subcutaneous abdominal fat mass was significantly associated with an increased risk (10). Meanwhile, previous studies showed conflicting results on the contribution of skeletal muscle mass to the pathogenesis of insulin resistance and metabolic status (14–17). Abdominal fat distribution and skeletal muscle mass vary significantly between racial and ethnic groups (18), however, few studies have investigated the abdominal fat distribution and skeletal muscle mass in different metabolic phenotypes in a population-based study with a large sample size in China.

The aim of this study was to investigate the characteristics of abdominal fat distribution and skeletal muscle measured by using quantitative computed tomography (QCT) in different metabolic status in Chinese participants. We explored whether higher abdominal visceral adipose tissue (VAT) and lower abdominal subcutaneous adipose tissue (SAT) and skeletal muscle index (SMI) were associated with an increased risk of metabolically unhealthy phenotype. We also assessed the ability of abdominal fat distribution and skeletal muscle mass to identify subjects with different metabolically phenotypes.

## Methods

### Study Design and Population

The population of the present cross-sectional study was retrieved from Pinggu Metabolic Disease Study (PMDS). Participants were recruited using a stratified random two-stage cluster sampling process according to sex and age. In the first stage of sampling, five rural towns and one street were first randomly selected. Five villages and seven neighborhood communities were then randomly drawn from each selected rural town and the street. In total, 5004 individuals who had lived in their registered address for >5 years were invited to participate, and 3350 accepted. In the second round, the 5004 originally selected residents were invited again to participate, and an additional 1579 residents were invited between September 2013 and July 2014 for a total of 6583 invited residents. A total of 4002 people aged 26–77 years were enrolled, which gave a response rate of 60.8%. Details including study enrollment and procedure about this study can be found elsewhere (19, 20). Participants with a BMI less than 18.5 kg/m^2^ (underweight) were excluded from our study. Finally, 3, 954 participants were included in our analysis.

Ethical approval was obtained from Peking University Health Science Center (ethical review approval number: 2021PHB441-001). All participants provided written informed consent before enrollment.

### Questionnaire and Physical Examination

All participants accepted face-to-face interview and standardized questionnaires as described in detail previously. Height and weight were measured using a height-weight scale that had been calibrated before use, with the subject standing on their bare feet and in light clothing. BMI was calculated as weight divided by height squared (kg/m^2^). Waist circumference (WC) was measured using a tape measure at the midpoint between the lower rib margin and the iliac crest to the nearest 0.1 cm. Hip circumference (HC) was measured at the midpoint of the iliac crest and the most lateral projecting points of the greater trochanter to the nearest 0.1 cm. The waist-to-hip ratio (WHR) was calculated as the ratio of waist circumference to hip circumference. Blood pressure (BP) was measured three times with subjects seated after at least 5 min of rest, the mean value of the three measurements was recorded.

### Biochemical and Hormone Assays

Fasting blood samples from participants were collected after 8-hour fasting. Fasting serum alanine aminotransferase (ALT), aspartate aminotransferase (AST), total cholesterol (TC), triglyceride (TG), high-density lipoprotein cholesterol (HDL-C), low-density lipoprotein cholesterol (LDL-C), and creatinine (Cr) levels and the uric acid (UA) were measured using the automated biochemical instrument (Coulter UniCel DxC 800, Beckman, Miami, FL, USA).

Participants without known diabetes underwent a 75g 2-h oral glucose tolerance test (OGTT) to evaluate the status of glucose tolerance and those with diabetes had fasting plasma glucose (FPG) measured. Plasma glucose was measured by hexokinase method. Hemoglobin A1c (HbA1c) was measured by cation-exchange high-pressure liquid chromatography (HPLC) method (Adams A1c HA-8160; Arkray, Kyoto, Japan). Serum insulin was tested by a radioimmunoassay method (China Institute of Atomic Energy, Beijing, China). Homeostasis model assessment of insulin resistance (HOMA-IR) was calculated as follows: fasting insulin (FINS) (mU/ml) × fasting glucose (mmol/l)/22.5.

### QCT Measurements

A routine abdominal plain CT scan was performed with a GE 64-slice CT scanner (LightSpeed VCT, GE, USA). The CT scan acquired continuous 5-mm thick slices (120 kVp, 120–150 mA) from the lung base to the pubic symphysis in the supine position.

The quantification of abdominal fat was detected at the level of the lumbar vertebrae 4–5 intervertebral disc space. Abdominal total adipose tissue (TAT) and visceral adipose tissue (VAT) area (cm^2^) were semi-automatically measured by the Tissue Composition Module of the software (Mindways, Austin, TX, USA). Subcutaneous adipose tissue (SAT) was calculated as TAT–VAT. The third lumbar vertebrae (L3) was selected as a standard landmark to measure skeletal muscle area (SMA). The L3 region contains the psoas, paraspinal, and abdominal wall muscles. The cross-sectional SMAs were measured according to attenuation thresholds of -29 to +150 Hounsfield units. We calculated skeletal muscle indexes (SMI; cm^2^/m^2^) by normalizing the total skeletal muscle surface area by the height in square metres. Abdominal fat was also detected at the level of L3, and skeletal muscle was also detected at the level of L4-5 using same methods as exploratory indicators.

### Definition

Subjects were classified as normal weight (18.5–23.9 kg/m^2^), overweight (24.0–27.9 kg/m^2^), and obese (≥28.0 kg/m^2^) according to BMI recommended by the Working Group on Obesity in China (21). The absence of the metabolic syndrome (MS) in obesity has commonly been used to define MHO. According to the diagnostic criteria for MS of the Chinese Diabetes Society (CDS) (22), participants who met <2 of the following 4 criteria were considered metabolically healthy: (1) FPG≥6.1mmol/L or 2hPG≥7.8mmol/L or taking anti-diabetic medications; (2) systolic blood pressure (SBP) ≥ 130 mmHg or diastolic blood pressure (DBP) ≥ 85 mmHg or a history of taking anti-hypertensive medication; (3) triglyceride (TG) ≥ 1.7 mmol/L; (4) high-density lipoprotein cholesterol (HDL-C) < 1.04 mmol/L or on any medication(s) for these conditions. According to previous related studies, waist circumference was not used to define these phenotypes due to its colinearity with BMI.

We divided participants into the following six groups on the basis of BMI and metabolic health status: (1) metabolically healthy normal weight (MHNW); (2) metabolically unhealthy normal weight (MUNW); (3) metabolically healthy overweight (MHOW); (4) metabolically unhealthy overweight (MUOW); (5) metabolically healthy obese (MHO); and (6) metabolically unhealthy obese (MUO).

### Statistical Analysis

Clinical characteristics were shown as mean ± standard deviation (SD) or numbers and percentages, as appropriate. Shapiro-Wilk tests were used to verify the normal or skewed distributions of continuous variables. Student’s t test was conducted to compare normally distributed continuous variables, while Mann–Whitney U test or Kruskal–Wallis H test were conducted to compare non-parametric continuous variables between different groups. Chi-squared test or Fisher’s exact test were performed to assess differences in categorical variables between groups. All participants were divided into tertiles based on the levels of above indices. Logistic regression analysis was employed to test the odds ratio (OR) and 95% confidence intervals (CI) of indices of body composition for metabolically unhealthy risks with the lowest group as the referent group after adjustment for age, sex and BMI. The receiver operating characteristic curve (ROC) analysis was used to explore the capability of indices of body composition, such as TAT, VAT, SAT, VAT and TAT ratio (at L4-5), SMI (at L3), SMA and TAT ratio (at L3 and L4-5) to differentiate metabolically unhealthy subjects from controls.

All statistical analysis were performed by SPSS version 23.0 software for windows (SPSS Inc., Chicago, IL, USA). A *p* value <0.05 was considered to be statistically significant.

## Results

### General Characteristics

Totally, 3, 954 participants with a mean age of 50.2 ± 11.7 years were included in final analysis. 63.4%, 39.5% and 23.3% participants with normal weight, overweight and obese were classified as metabolically healthy, respectively. The levels of BMI, WC, WHR, FPG, FINS, HbA1c, HOMA-IR, SBP, DBP, cholesterol, and triglyceride were higher, the levels of HDL-C were lower in participants with metabolically unhealthy phenotypes (MUNW, WUOW and MUO) (all *p <*0.001), when compared with participants with metabolically healthy phenotypes (MHNW, MHOW and MHO). In participants with overweight and obese, the levels of LDL-C were similar between metabolically healthy and unhealthy groups (*p*=0.178 and 0.664, respectively). Individuals with metabolically unhealthy phenotype had a higher proportion of individuals with non-alcoholic fatty liver disease (NAFLD) and diabetes. More men were included into metabolically unhealthy phenotypes (**Table 1**).

**Table 1:** Demographic and clinical characteristics of study population.

		Normal weight			Overweight			Obese			
Variables	Total N=3954	Metabolically healthy N=742	Metabolically unhealthy N=428	*P* value	Metabolically healthy N=647	Metabolically unhealthy N=989	*P* value	Metabolically healthy N=267	Metabolically unhealthy N=880	*P* value
Age (years)	50.2 ± 11.7	47.4 ± 12.4	53.3 ± 11.9	***<0.001***	48.7 ± 11.4	52.8 ± 10.7	***<0.001***	46.7 ± 10.7	50.6 ± 11.5	***<0.001***
Men (%)	49.1	38.7	62.9	***<0.001***	41.9	55.7	***<0.001***	36.0	53.2	***<0.001***
Annual household income (n, %)										
<¥50,000	1988 (50.3)	370 (49.9)	245 (57.2)	***0.015***	334 (51.6)	482 (48.7)	0.266	146 (54.7)	411 (46.7)	***0.021***
¥50,000–99,000	1426 (36.1)	280 (37.8)	133 (31.0)	***<0.001***	226 (34.9)	365 (36.8)	0.461	91 (34.0)	331 (37.6)	0.311
≥¥100,000	540 (13.7)	92 (12.4)	50 (11.7)	0.781	87 (13.5)	143 (14.4)	0.662	30 (11.2)	138 (15.7)	0.076
Education (n, %)										
Elementary school or lower	750 (19)	121 (16.3)	117 (27.3)	***<0.001***	92 (14.2)	197 (19.9)	***0.003***	31 (11.6)	192 (21.8)	***<0.001***
Middle school	2601 (65.8)	475 (64.0)	265 (62.0)	0.529	446 (68.9)	652 (65.9)	0.216	191 (71.6)	572 (65.0)	0.054
College or higher	603 (15.3)	146 (19.7)	46 (10.7)	***<0.001***	109 (16.8)	141 (14.2)	0.160	45 (16.9)	116 (13.2)	0.132
BMI (kg/m2)	26.21 ± 3.74	21.93 ± 1.38	22.34 ± 1.28	***<0.001***	25.79 ± 1.12	26.10 ± 1.12	***<0.001***	30.40 ± 2.35	30.86 ± 2.57	***0.003***
WC (cm)	87.0 ± 10.6	75.1 ± 6.1	78.9 ± 5.7	***<0.001***	84.5 ± 6.2	88.5 ± 6.0	***<0.001***	94.7 ± 7.7	98.9 ± 7.5	***<0.001***
HC (cm)	98.5 ± 7.1	92.4 ± 4.7	92.6 ± 4.8	0.788	97.9 ± 4.5	98.3 ± 4.5	0.300	105.0 ± 6.3	105.4 ± 6.0	***<0.001***
WHR	0.88 ± 0.07	0.81 ± 0.06	0.85 ± 0.05	***<0.001***	0.86 ± 0.06	0.90 ± 0.06	***<0.001***	0.90 ± 0.06	0.94 ± 0.06	***<0.001***
NAFLD (n, %)	88.2 (22.3)	75 (10.1)	73 (17.1)	***<0.001***	76 (11.7)	252 (25.5)	***<0.001***	50 (18.7)	356 (40.5)	***<0.001***
ALT (U/L)	23.9 ± 18.6	18.5 ± 11.0	22.3 ± 36.4	***<0.001***	22.0 ± 13.2	24.6 ± 14.4	***<0.001***	22.4 ± 10.6	30.3 ± 18.7	***<0.001***
AST (U/L)	23.2 ± 11.4	21.5 ± 8.1	23.7 ± 18.6	***<0.001***	22.7 ± 10.8	22.9 ± 9.1	0.201	21.4 ± 6.0	25.4 ± 12.9	***<0.001***
Cr (μmol/L)	61.5 ± 25.7	57.6 ± 26.6	65.3 ± 46.7	***<0.001***	58.8 ± 12.6	64.0 ± 28.0	***<0.001***	58.5 ± 13.6	63.3 ± 14.7	***<0.001***
UA (μmol/L)	285.9 ± 80.5	246.5 ± 60.9	283.7 ± 79.4	***<0.001***	272.7 ± 76.2	297.0 ± 79.0	***<0.001***	278.1 ± 74.7	319.9 ± 85.1	***<0.001***
Glucose tolerance										
Normal (n, %)	2172 (54.9)	667 (89.9)	147 (34.3)	***<0.001***	564 (88.7)	311 (31.4)	***<0.001***	238 (89.1)	235 (26.7)	***<0.001***
IGT/IFG (n, %)	1154 (29.2)	57 (7.7)	194 (45.3)	***<0.001***	52 (8.0)	442 (44.6)	***<0.001***	21 (7.9)	388 (44.1)	***<0.001***
Diabetes (n, %)	628 (15.9)	18 (2.4)	87 (20.3)	***<0.001***	21 (3.2)	237 (23.9)	***<0.001***	8 (3.0)	257 (29.2)	***<0.001***
FPG (mmol/L)	6.09 ± 1.64	5.41 ± 0.91	6.46 ± 2.28	***<0.001***	5.5 ± 0.8	6.5 ± 1.7	***<0.001***	5.55 ± 0.68	6.64 ± 1.91	***<0.001***
FINS (uIU/ml)	9.78 ± 6.61	5.82 ± 2.83	6.95 ± 6.06	***<0.001***	7.82 ± 3.67	10.44 ± 6.18	***<0.001***	11.07 ± 5.03	14.79 ± 8.09	***<0.001***
HbA1c (%)	5.84 ± 0.94	5.49 ± 0.64	5.91 ± 1.16	***<0.001***	5.52 ± 0.44	6.00 ± 1.02	***<0.001***	5.58 ± 0.43	6.21 ± 1.11	***<0.001***
HOMA-IR	2.74 ± 2.39	1.40 ± 0.72	2.02 ± 2.44	***<0.001***	1.94 ± 1.06	3.04 ± 2.30	***<0.001***	2.77 ± 1.51	4.45 ± 3.10	***<0.001***
SBP (mmHg)	130 ± 18	120 ± 10	134 ± 19	***<0.001***	125 ± 15	135 ± 17	***<0.001***	127 ± 16	137 ± 17	***<0.001***
DBP (mmHg)	79 ± 11	72 ± 10	79 ± 11	***<0.001***	76 ± 10	81 ± 11	***<0.001***	78 ± 10	84 ± 11	***<0.001***
Cholesterol (mmol/l)	4.93 ± 0.99	4.70 ± 0.84	5.00 ± 1.10	***<0.001***	4.89 ± 0.90	4.97 ± 0.99	***0.042***	4.81 ± 0.80	5.11 ± 1.09	***<0.001***
Triglyceride (mmol/l)	1.60 ± 1.47	0.81 ± 0.44	1.64 ± 1.63	***<0.001***	0.99 ± 0.51	2.02 ± 1.63	***<0.001***	1.10 ± 0.46	2.39 ± 1.81	***<0.001***
LDL-C (mmol/l)	2.88 ± 0.81	2.70 ± 0.71	2.86 ± 0.87	***0.002***	2.96 ± 0.80	2.89 ± 0.82	0.178	2.98 ± 0.69	2.96 ± 0.88	0.664
HDL-C (mmol/l)	1.16 ± 0.31	1.36 ± 0.29	1.19 ± 0.40	***<0.001***	1.27 ± 0.29	1.06 ± 0.24	***<0.001***	1.19 ± 0.29	1.01 ± 0.24	***<0.001***

Data are expressed as means ± SD for continuous data or as n (%) for categorical data. Bold italics indicate statistical differences. WC, Waist circumference; HC, Hip circumference; WHR, waist-to-hip ratio; FPG, Fasting plasma glucose; FINS, Fasting insulin; HOMA-IR, homeostasis model assessment of insulin resistance.

### Abdominal Fat Distribution and Skeletal Muscle in Different Metabolically Phenotypes

**Figure 1:**
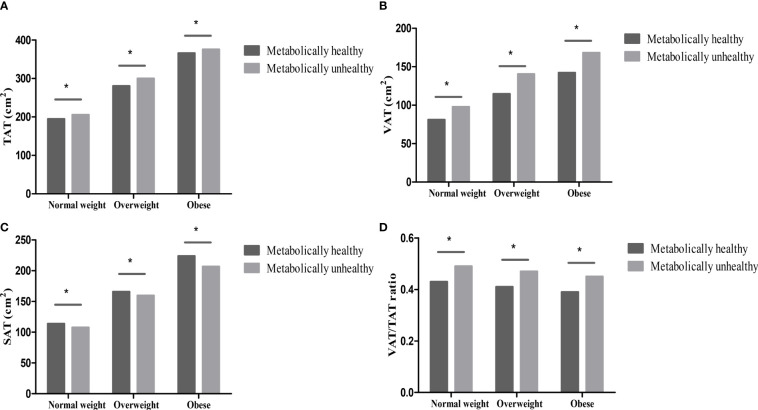
Abdominal fat distribution in different metabolically phenotypes. **(A)** TAT in different metabolically phenotypes. **(B)** VAT in different metabolically phenotypes. **(C)** SAT in different metabolically phenotypes. **(D)** VAT/TAT ratio in different metabolically phenotypes. *p value < 0.05.

**Figure 2:**
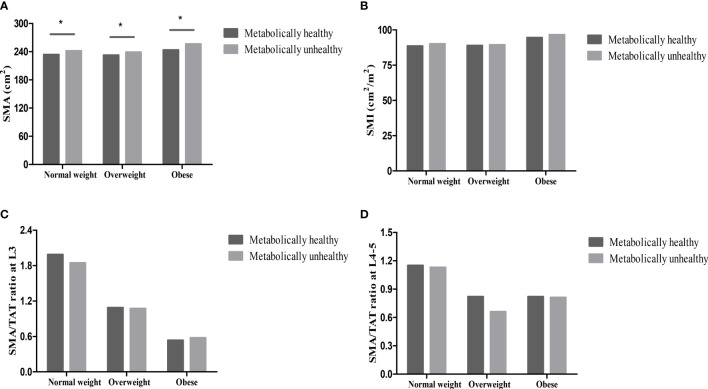
Skeletal muscle in different metabolically phenotypes. **(A)** SMA in different metabolically phenotypes. **(B)** SMI in different metabolically phenotypes. **(C)** SMA/TAT ratio at L3 in different metabolically phenotypes. **(D)** SMA/TAT ratio at L4-5 in different metabolically phenotypes. *p value<0.05.

Compared with individuals with metabolically unhealthy phenotypes, the levels of abdominal TAT, VAT, VAT/TAT ratio, and SMA were lower, while the levels of SAT were higher in individuals with metabolically healthy phenotypes (all *p <*0.001) in different BMI categories (normal weight, overweight, and obese). However, the levels of SMI and SMA/TAT ratio were similar in metabolically healthy and unhealthy groups (all *p*>0.05) (**Figures 1**, **2**).

We further analyzed the abdominal fat distribution and skeletal muscle in males and females, respectively. The levels of TAT, VAT, and SAT were higher in MUNW group than that in MHNW group in both men and women (all *p <*0.001). However, the levels of SMI were lower in MUNW group than that in MHNW group in women (*p*=0.003) but not in men (*p*=0.275). The levels of TAT, and VAT were higher in both men and women, while the levels of SAT were only higher in men in MUOW group than that in MHOW group (all *p <*0.001). There were no significant difference of SMI between the two groups (both *p*>0.05). The levels of VAT were higher in both men and women, while the levels of TAT were only higher in men with MUO phenotype than that with MHO phenotype (all *p <*0.001). However, the levels of SAT was higher in women in individuals with MHO phenotype when compared with MUO phenotype (*p*=0.016). There were no significant difference of SMI between the two groups (both *p*>0.05) (**Supplemental Table 1**).

### Associations of Abdominal Fat Distribution and Skeletal Muscle With Metabolic Status

In individuals with normal weight, the highest abdominal TAT, VAT, and VAT/TAT ratio category were associated with a 2.093 times (95%CI: 1.043-3.123), 2.669 times (95%CI: 1.864-3.821), and 2.077 times (95%CI: 1.345-3.209) higher risk of MUNW when compared with the lowest category, respectively (all *p*<0.01). Risk of MUNW was reduced significantly in the highest SMA/TAT ratio category (L3: OR 0.437, 95%CI: 0.290-0.658, *p*<0.001; L4-5: OR 0.498, 95%CI: 0.323-0.767, *p*=0.002) when compared with the lowest category.

In individuals with overweight, the highest abdominal TAT, VAT, and VAT/TAT ratio category were associated with a 1.739 times (95%CI: 1.295-2.335), 3.275 times (95%CI: 2.427-4.421), and 2.676 times (95%CI: 1.925-3.720) higher risk of MUOW when compared with the lowest category, respectively (all *p*<0.01). Risk of MUOW was reduced significantly in the highest SMA/TAT ratio category (L3: OR 0.505, 95%CI: 0.369-0.689, *p*<0.001; L4-5: OR 0.681, 95%CI: 0.486-0.953, *p*=0.025) when compared with the lowest category.

In individuals with obese, the highest abdominal VAT, VAT/TAT ratio, and SMA/TAT ratio category were associated with a 2.322 times (95%CI: 1.469-3.670), 3.187 times (95%CI: 2.024-5.019), and 1.640 (95%CI: 1.024-2.626) times higher risk of metabolically unhealthy when compared with the lowest category, respectively (all *p*<0.05). While, in individuals with obese, risk of MUO was reduced significantly in the highest SAT category (OR=0.555, 95%CI: 0.360-0.856, *p*=0.008) when compared with the lowest category. However, the levels of SMI did not have significant association with metabolic status in different BMI categories (**Table 2**).

**Table 2:** Odds ratio of abdominal fat distribution and skeletal muscle category for metabolically unhealthy according to BMI category.

	Normal weight		Overweight		Obese	
	OR, 95%CI	*p* value	OR, 95%CI	*p* value	OR, 95%CI	*p* value
TAT at L4-5						
Lowest tertile	1.00 (reference)		1.00 (reference)		1.00 (reference)	
Median tertile	1.591 (1.114, 2.272)	***0.011***	1.519 (1.160, 1.989)	***0.002***	0.817 (0.561, 1.189)	0.290
Highest tertile	2.093 (1.043, 3.123)	***<0.001***	1.739 (1.295, 2.335)	***<0.001***	1.298 (0.838, 2.011)	0.243
VAT at L4-5						
Lowest tertile	1.00 (reference)		1.00 (reference)		1.00 (reference)	
Median tertile	1.491 (1.052, 2.113)	***0.025***	1.669 (1.283, 2.169)	***<0.001***	1.258 (0.877, 1.805)	0.213
Highest tertile	2.669 (1.864, 3.821)	***<0.001***	3.275 (2.427, 4.421)	***<0.001***	2.322 (1.469, 3.670)	***<0.001***
SAT at L4-5						
Lowest tertile	1.00 (reference)		1.00 (reference)		1.00 (reference)	
Median tertile	1.055 (0.736, 1.511)	0.772	0.875 (0.660, 1.161)	0.355	0.792 (0.527, 1.191)	0.262
Highest tertile	1.192 (0.766, 1.857)	0.436	0.805 (0.582, 1.113)	0.189	0.555 (0.360, 0.856)	***0.008***
VAT/TAT at L4-5						
Lowest tertile	1.00 (reference)		1.00 (reference)		1.00 (reference)	
Median tertile	1.556 (1.083, 2.237)	***0.017***	1.588 (1.200, 2.101)	***0.001***	2.261 (1.546, 3.306)	***<0.001***
Highest tertile	2.077 (1.345, 3.209)	***0.001***	2.676 (1.925, 3.720)	***<0.001***	3.187 (2.024, 5.019)	***<0.001***
SMI at L3						
Lowest tertile	1.00 (reference)		1.00 (reference)		1.00 (reference)	
Median tertile	0.946 (0.679, 1.318)	0.743	0.965 (0.736, 1.265)	0.797	0.779 (0.527, 1.151)	0.210
Highest tertile	0.705 (0.493, 1.007)	0.705	0.897 (0.673, 1.195)	0.457	0.980 (0.634, 1.517)	0.929
SMA/TAT at L3						
Lowest tertile	1.00 (reference)		1.00 (reference)		1.00 (reference)	
Median tertile	0.604 (0.427, 0.853)	***0.004***	0.875 (0.660, 1.159)	0.352	0.903 (0.597, 1.368)	0.631
Highest tertile	0.437 (0.290, 0.658)	***<0.001***	0.505 (0.369, 0.689)	***<0.001***	0.683 (0.430, 1.084)	0.106
SMA/TAT at L4-5						
Lowest tertile	1.00 (reference)		1.00 (reference)		1.00 (reference)	
Median tertile	0.555 (0.385, 0.801)	***0.002***	1.124 (0.841, 1.502)	0.430	1.189 (0.794, 1.782)	0.401
Highest tertile	0.498 (0.323, 0.767)	***0.002***	0.681 (0.486, 0.953)	***0.025***	1.640 (1.024, 2.626)	***0.040***

Bold italics indicate statistical differences. TAT, total adipose tissue; VAT, visceral adipose tissue; SAT, subcutaneous adipose tissue; SMI, skeletal muscle index. Adjusted for age, sex and BMI.

### Diagnostic Values of Indices of Body Composition for Metabolically Unhealthy

We assessed the diagnostic values of indices of body composition, such as abdominal TAT, VAT, SAT, VAT and TAT ratio (at L4-5), SMI (at L3), SMA and TAT ratio (at L3 and L4-5), for metabolically unhealthy (**Table 3**). In individuals with normal weight, VAT and VAT/TAT ratio were the two best indicators to differentiate MHNW and MUNW with the area under ROC curve (AUC) of 0.653 (95% CI: 0.619-0.687, *p*<0.001), and 0.652 (95% CI: 0.620-0.685, *p*<0.001). The optimal cutoff value for VAT and VAT/TAT ratio were 89.23 cm^2^, and 0.455, respectively. The sensitivity and specificity were 59.11% and 67.28% for VAT, and 64.04% and 62.36% for VAT/TAT ratio.

**Table 3:** ROC curves of indices of body composition for metabolically unhealthy.

	Normal weight		Overweight		Obese	
	AUC (95%CI)	*p* value	AUC (95%CI)	*p* value	AUC (95%CI)	*p* value
TAT	0.547 (0.512, 0.583)	***0.009***	0.574 (0.544, 0.605)	***<0.001***	0.546 (0.503, 0.590)	***0.045***
VAT	0.653 (0.619, 0.687)	***<0.001***	0.682 (0.654, 0.711)	***<0.001***	0.668 (0.627, 0.710)	***<0.001***
SAT	0.537 (0.502, 0.572)	***0.005***	0.549 (0.518, 0.580)	***0.002***	0.583 (0.627, 0.539)	***<0.001***
VAT/TAT ratio	0.652 (0.620, 0.685)	***<0.001***	0.651 (0.622, 0.680)	***<0.001***	0.687 (0.645, 0.728)	***<0.001***
SMI	0.530 (0.494, 0.565)	1.000	0.504 (0.474, 0.535)	0.775	0.536 (0.491, 0.580)	0.122
SMA/TAT ratio at L3	0.553 (0.518, 0.589)	***0.003***	0.565 (0.535, 0.596)	***<0.001***	0.523 (0.479, 0.567)	0.321
SMA/TAT ratio at L4-5	0.513 (0.477, 0.527)	0.473	0.509 (0.478, 0.540)	0.573	0.532 (0.487, 0.577)	0.162

TAT, total adipose tissue; VAT, visceral adipose tissue; SAT, subcutaneous adipose tissue; SMI, skeletal muscle index.

In individuals with overweight, abdominal VAT and VAT/TAT ratio were the two best indicators to differentiate MHOW and MUOW with the AUC of 0.682 (95% CI: 0.654-0.711, *p*<0.001), and 0.651 (95% CI: 0.622-0.680, *p*<0.001). The optimal cutoff value for VAT and VAT/TAT ratio were 127.3 cm^2^, and 0.455, respectively. The sensitivity and specificity were 59.24% and 67.91% for VAT, and 57.13% and 64.53% for VAT/TAT ratio.

**Figure 3:**
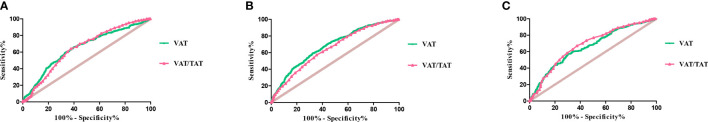
ROC curves of VAT and VAT/TAT ratio for metabolically unhealthy in individuals with normal weight **(A)**, overweight **(B)**, and obese **(C)**.

In individuals with obese, VAT/TAT ratio and VAT were the two best indicators to differentiate MHO and MUO with the AUC of 0.687 (95% CI: 0.645-0.728, *p*<0.001), and 0.668 (95% CI: 0.627-0.710, *p*<0.001), respectively. The optimal cutoff value for VAT/TAT ratio and VAT were 0.415, and 155.8 cm^2^, respectively. The sensitivity and specificity were 67.11% and 62.61% for VAT/TAT ratio, and 57.56% and 69.57% for VAT (**Figure 3**).

## Discussion

In this population-based study using CT techniques to ascertain body composition and its relation with metabolically healthy status in a large sample of Chinese population, we found 63.4%, 39.5%, and 23.3% participants were classified as MHNW, MHOW, and MHO, respectively. Higher levels of abdominal visceral adipose tissue were closely associated with metabolically unhealthy phenotype in Chinese adults with different BMI categories. Abdominal subcutaneous adipose tissue might be a protective factor for metabolic health status only in obese individuals. However, there was no significant association between the levels of SMI and metabolic status in different BMI categories.

The mechanisms underlying metabolic health status are complex. The different impact of specific fat compartments on insulin sensitivity and lipid levels was well described, with excess abdominal VAT imparting a greater risk of metabolic syndrome than excess abdominal SAT. Visceral adipose tissue acted not only as a fat-deposit site, but also as a highly secretory organ with a differential production of adipokines capable of regulating lipid metabolism, insulin sensitivity, and inflammation (23). Previous studies found MHO individuals had significantly lower levels of visceral fat than postmenopausal women with MUO phenotype (7, 24). In our study, we used QCT, an accurate method, to assess abdominal fat area, and we found abdominal VAT was closely associated with metabolically unhealthy phenotype in different BMI categories. These findings supported the opinion that a smaller amount of abdominal VAT was a protective factor in the maintenance of the favorable metabolic profile.

Some evidence indicated ectopic fat accumulation was a plausible mechanism for metabolic syndrome (25). The liver was an important location for ectopic fat deposition. Norbert Stefan et al. found ectopic fat in the liver may be more important than visceral fat in the determination of a beneficial phenotype in obesity (13). Though we did not assess ectopic fat accumulation in the liver, lower concentrations of hepatic enzymes in metabolically healthy individuals may reflect lower hepatic insulin resistance and lower liver fat content (23). However, we also need to emphasize that liver fat content is high but insulin resistance and the risk of diabetes are not in patients with fatty liver disease with a strong hepatic genetic component (ie, the 148Met allele in *PNPLA3* and the 167Lys allele in *TM6SF2*) (26, 27). Future studies will be required to unravel underlying mechanisms of ectopic fat accumulation and its contribution to metabolic unhealthy phenotype in Chinese population.

Massive expansion and remodeling of adipose tissue during obesity differentially affects specific adipose tissue depots and significantly contributes to metabolic unhealth status (28). Subcutaneous adipocytes have distinctly different gene expression patterns (higher adiponectin expression and lower expression of proinflammatory adipokines), are better differentiated, and have increased adipogenesis and browning potential compared with visceral adipocytes (29, 30). In most obese individuals, the SAT might fail to expand to store energy surplus, and lipids might accumulate ectopically in VAT, liver, and skeletal muscle; whereas in healthy obese individuals, SAT had the intrinsic ability to expand, leading to preserved insulin sensitivity (31, 32). Observations of Framingham Heart Study showed that the inability to store fat in subcutaneous adipose tissue depot increases the propensity for visceral fat storage (33). It was found that regulation of lipid storage-related genes was defective in the SAT of subjects exhibiting the largest fat accumulation in the VAT of non-obese subjects when overfed for 56 days (32). In our study, abdominal SAT might be a protective factor for MHO, which might be explained by a greater ability of MHO individuals to store free-fatty acids in the SAT instead of in ectopic fat depot. However, the levels of abdominal SAT were similar in metabolically healthy and metabolically unhealthy individuals with normal weight and overweight. This finding was not consistent with previous studies in Caucasians. A low percentage subcutaneous leg fat mass can be found with high prevalence in both unhealthy normal weight subjects and overweight and obese subjects (12). In addition, low percentage leg fat mass, followed by fatty liver, is the strongest independent predictor of metabolic risk in normal weight subjects, however, it is not a significant determinant of metabolic risk in obese subjects (12). The associations between subcutaneous fat at different sites and metabolic status warrant further investigation.

Muscle mass and strength were protective factors against cardiometabolic risk and metabolic syndrome, although the conclusions were inconsistent (34, 35). Protective effects of muscle mass were likely due to mechanisms involving glucose utilization, myokines secretion and ectopic fat accumulation (36, 37). However, in this study, we did not find significant association between the levels of SMI and metabolic status. In addition, muscle and fat ratio (SMA/TAT ratio) was not a more useful indicator than VAT or VAT/TAT ratio for screening metabolic syndrome. Further studies on potential associations and mechanisms underlying skeletal muscle and metabolic status were needed.

In our study, we found MHO individuals represent 23.3% of the adult obese population, with higher prevalence in women, which was similar to previous studies (38–40). Though, MHO individuals present a favorable blood lipid profile, favorable hepatic enzyme profile, lower blood pressure and lower insulin resistance compared with MUO individuals, MHO individuals have an intermediate-stage of cardiovascular risk profile that is between MUO and non-obese phenotypes, and might shift to an MUO phenotype with time (41–43). Hwang et al. demonstrated that a higher conversion to MUO was associated with greater visceral abdominal fat, female gender, higher fasting insulin levels, and lower baseline HDL-C levels (44). Some researchers even suggested that using the label ‘metabolically healthy’ to describe this group in clinical medicine might be misleading (43). Therefore, a more profound understanding of the underlying metabolic regulation in MHO and MUO needed further research. Importantly, it was necessary to encourage exercise and weight control for MHO population to prevent metabolic related disorders due to its transitory state.

Our findings had important clinical implications and public health importance. We found that patients within the same BMI range had heterogeneous phenotypes. We also found that obese subjects or normal weight subjects ran different risks of complications. Therefore, stratification of individuals, based on metabolic health status, would help to identify high-risk subjects and to optimize prevention and treatment strategies in order to bring benefits to the individual, and lessen the burden on the health care system. According to this study, the analysis of ROC curves demonstrated that abdominal VAT and VAT/TAT ratio might help to predict metabolic healthy status. It meant that patients with normal weight and overweight might have metabolic syndrome if the VAT and VAT/TAT ratio exceeded cut-off levels, and screening interim might be shortened for risk prevention of complications in these patients. More importantly, there might be metabolism healthy subjects if the VAT and VAT/TAT ratio was below cut-off levels for obese individuals. In these subjects, the intensity of treatment and the frequency of screening might be weakened.

There were several strengths of our study. Firstly, this was the first large population-based study focusing on the associations of adipose distributions and muscle mass measured by QCT with the relation of metabolic status in different BMI category among Chinese adults. Secondly, most previous studies used waist to height ratio, waist to hip ratio or indices measured by bioelectrical impedance analyzer or dual-energy x-ray absorptiometry as measures of body composition (23, 45). In our study, we used QCT, which was considered as the gold standard for quantifying and comparing regional fat distribution and skeletal muscle. Thirdly, most previous studies evaluated characteristic of MHO and MUO (41). In our study, we evaluated not only obese individuals, but also normal weight and overweight participants according to metabolic status.

However, our study has several limitations. Firstly, given the inconsistency of metabolically healthy definitions, there was high degree of variability surrounding the estimated prevalence of different phenotypes. Secondly, we did not measure hepatic fat content, and only used serum activities of hepatic enzymes to represent hepatic fat content. Gluteofemoral fat mass had effects on metabolic health status, however, we did not measure lower-body fat mass in our study. In addition, we measured skeletal muscle mass without considering the muscle quality. Good-quality muscle and poor-quality muscle might have different contributions to metabolic status. Thirdly, chronic or acute diseases might influence BMI and metabolic health status. However, comorbidity and associated pharmacological treatment were not described in detail in this study. Fourthly, human obesity is associated with an imbalance in adipokines, which could act as classic hormones affecting the metabolism of tissues and organs. What is more, adipokines may decrease the insulin sensitivity of tissues and induce inflammation and development of chronic complications. However, we did not measure adipokines in our study. Fifthly, the cross-sectional design did not allow us to elucidate the causal relationship between abdominal fat distribution and skeletal muscle and the metabolically phenotypes.

In conclusion, metabolically healthy individuals were relatively protected against metabolic disease as compared to metabolically unhealthy individuals, which was at least partly due to a better adipose tissue function and less ectopic fat storage. Measurement of abdominal fat distribution and skeletal muscle may provide a more complete understanding of metabolic risk.

## Data Availability Statement

The original contributions presented in the study are included in the article/**Supplementary Material**. Further inquiries can be directed to the corresponding authors.

## Ethics Statement 

The studies involving human participants were reviewed and approved by Peking University Health Science Center. Written informed consent for participation was not required for this study in accordance with the national legislation and the institutional requirements.

## Author Contributions

LJ and XC conceptualized this study and designed the systematic review protocol. FL, YL, XYZ, XH, XHZ, and ZF performed the study selection and data extraction. FL and XC performed the statistical analyses. FL and XC prepared the outlines and wrote the manuscript. All authors contributed to the critical revision of manuscript drafts.

## Funding

This work was supported by National Natural Science Foundation of China (No.81970698, No.81970708, and No.81900805), and Beijing Natural Science Foundation (No.7202216). The funding agencies had no roles in the study design, data collection or analysis, decision to publish or preparation of the manuscript.

## Conflict of Interest

The authors declare that the research was conducted in the absence of any commercial or financial relationships that could be construed as a potential conflict of interest.

## Publisher’s Note

All claims expressed in this article are solely those of the authors and do not necessarily represent those of their affiliated organizations, or those of the publisher, the editors and the reviewers. Any product that may be evaluated in this article, or claim that may be made by its manufacturer, is not guaranteed or endorsed by the publisher.

## References

[1] Piché ME Tchernof A Després JP. Obesity Phenotypes, Diabetes, and Cardiovascular Diseases. Circ Res (2020) 126:1477–500. doi: 10.1161/CIRCRESAHA.120.316101 32437302

[2] Wildman RP Muntner P Reynolds K McGinn AP Rajpathak S Wylie-Rosett J. The Obese Without Cardiometabolic Risk Factor Clustering and the Normal Weight With Cardiometabolic Risk Factor Clustering: Prevalence and Correlates of 2 Phenotypes Among the US Population (NHANES 1999-2004). Arch Intern Med (2008) 168:1617–24. doi: 10.1001/archinte.168.15.1617 18695075

[3] Phillips CM. Metabolically Healthy Obesity: Definitions, Determinants and Clinical Implications. Rev Endocr Metab Disord (2013) 14:219–27. doi: 10.1007/s11154-013-9252-x 23928851

[4] Schulze MB. Metabolic Health in Normal-Weight and Obese Individuals. Diabetologia (2019) 62:558–66. doi: 10.1007/s00125-018-4787-8 30569272

[5] Goossens GH. The Metabolic Phenotype in Obesity: Fat Mass, Body Fat Distribution, and Adipose Tissue Function. Obes Facts (2017) 10:207–15. doi: 10.1159/000471488 PMC5644968 28564650

[6] Karelis AD Faraj M Bastard JP St-Pierre DH Brochu M Prud’homme D. The Metabolically Healthy But Obese Individual Presents a Favorable Inflammation Profile. J Clin Endocrinol Metab (2005) 90:4145–50. doi: 10.1210/jc.2005-0482 15855252

[7] Messier V Karelis AD Robillard ME Bellefeuille P Brochu M Lavoie JM. Metabolically Healthy But Obese Individuals: Relationship With Hepatic Enzymes. Metabolism (2010) 59:20–4. doi: 10.1016/j.metabol.2009.06.020 19709695

[8] Chen P Hou X Hu G Wei L Jiao L Wang H. Abdominal Subcutaneous Adipose Tissue: A Favorable Adipose Depot for Diabetes? Cardiovasc Diabetol (2018) 17:93. doi: 10.1186/s12933-018-0734-8 29945626 PMC6020307

[9] Sam S. Differential Effect of Subcutaneous Abdominal and Visceral Adipose Tissue on Cardiometabolic Risk. Horm Mol Biol Clin Investig (2018) 33. doi: 10.1515/hmbci-2018-0014 29522417

[10] Neeland IJ Turer AT Ayers CR Powell-Wiley TM Vega GL Farzaneh-Far R. Dysfunctional Adiposity and the Risk of Prediabetes and Type 2 Diabetes in Obese Adults. JAMA (2012) 308:1150–9. doi: 10.1001/2012.jama.11132 PMC3556508 22990274

[11] Stefan N. Causes, Consequences, and Treatment of Metabolically Unhealthy Fat Distribution. Lancet Diabetes Endocrinol (2020) 8:616–27. doi: 10.1016/S2213-8587(20)30110-8 32559477

[12] Stefan N Schick F Häring HU. Causes, Characteristics, and Consequences of Metabolically Unhealthy Normal Weight in Humans. Cell Metab (2017) 26:292–300. doi: 10.1016/j.cmet.2017.07.008 28768170

[13] Stefan N Kantartzis K Machann J Schick F Thamer C Rittig K. Identification and Characterization of Metabolically Benign Obesity in Humans. Arch Intern Med (2008) 168:1609–16. doi: 10.1001/archinte.168.15.1609 18695074

[14] Kim HK Lee MJ Kim EH Bae SJ Kim KW Kim CH. Comparison of Muscle Mass and Quality Between Metabolically Healthy and Unhealthy Phenotypes. Obesity (Silver Spring) (2021) 29:1375–86. doi: 10.1002/oby.23190 34235892

[15] Kim G Lee SE Jun JE Lee YB Ahn J Bae JC. Increase in Relative Skeletal Muscle Mass Over Time and Its Inverse Association With Metabolic Syndrome Development: A 7-Year Retrospective Cohort Study. Cardiovasc Diabetol (2018) 17:23. doi: 10.1186/s12933-018-0659-2 29402279 PMC5798183

[16] Ko BJ Chang Y Jung HS Yun KE Kim CW Park HS. Relationship Between Low Relative Muscle Mass and Coronary Artery Calcification in Healthy Adults. Arterioscler Thromb Vasc Biol (2016) 36:1016–21. doi: 10.1161/ATVBAHA.116.307156 27034471

[17] Kim G Lee SE Lee YB Jun JE Ahn J Bae JC. Relationship Between Relative Skeletal Muscle Mass and Nonalcoholic Fatty Liver Disease: A 7-Year Longitudinal Study. Hepatology (2018) 68:1755–68. doi: 10.1002/hep.30049 29679374

[18] Katzmarzyk PT Bray GA Greenway FL Johnson WD Newton RL Jr Ravussin E. Racial Differences in Abdominal Depot-Specific Adiposity in White and African American Adults. Am J Clin Nutr (2010) 91:7–15. doi: 10.3945/ajcn.2009.28136 19828714 PMC6443262

[19] Hu P Li Y Zhou X Zhang X Zhang F Ji L. Association Between Physical Activity and Abnormal Glucose Metabolism-A Population-Based Cross-Sectional Study in China. J Diabetes Complications (2018) 32:746–52. doi: 10.1016/j.jdiacomp.2018.05.021 30017433

[20] Li Y Zhao Z Wang L Fu Z Ji L Wu X. The Prevalence of Osteoporosis Tested by Quantitative Computed Tomography in Patients With Different Glucose Tolerances. J Clin Endocrinol Metab (2020) 105:dgz036. doi: 10.1210/clinem/dgz036 31545362

[21] Chen C Lu FC Department of Disease Control Ministry of Health, PR China. The Guidelines for Prevention and Control of Overweight and Obesity in Chinese Adults. BioMed Environ Sci (2004) 17 Suppl:1–36. 15807475

[22] Weng J. Evolution in the Chinese Diabetes Society Standards of Care for Type 2 Diabetes. Diabetes Metab Res Rev (2016) 32:440–1. doi: 10.1002/dmrr.2826 27464264

[23] Xia L Dong F Gong H Xu G Wang K Liu F. Association Between Indices of Body Composition and Abnormal Metabolic Phenotype in Normal-Weight Chinese Adults. Int J Environ Res Public Health (2017) 14:391. doi: 10.3390/ijerph14040391 PMC5409592 28387701

[24] Messier V Karelis AD Prud’homme D Primeau V Brochu M Rabasa-Lhoret R. Identifying Metabolically Healthy But Obese Individuals in Sedentary Postmenopausal Women. Obesity (Silver Spring) (2010) 18:911–7. doi: 10.1038/oby.2009.364 19851302

[25] Gonçalves CG Glade MJ Meguid MM. Metabolically Healthy Obese Individuals: Key Protective Factors. Nutrition (2016) 32:14–20. doi: 10.1016/j.nut.2015.07.010 26440861

[26] Stefan N Cusi K. A Global View of the Interplay Between Non-Alcoholic Fatty Liver Disease and Diabetes. Lancet Diabetes Endocrinol (2022) 10:284–96. doi: 10.10.16/S2213-8587(22)00003-1 35183303

[27] Stefan N Häring HU Cusi K. Non-Alcoholic Fatty Liver Disease: Causes, Diagnosis, Cardiometabolic Consequences, and Treatment Strategies. Lancet Diabetes Endocrinol (2019) 7:313–24. doi: 10.1016/S2213-8587(18)30154-2 30174213

[28] Koenen M Hill MA Cohen P Sowers JR. Obesity, Adipose Tissue and Vascular Dysfunction. Circ Res (2021) 128:951–68. doi: 10.1161/CIRCRESAHA.121.318093 PMC8026272 33793327

[29] Estève D Boulet N Belles C Zakaroff-Girard A Decaunes P Briot A. Lobular Architecture of Human Adipose Tissue Defines the Niche and Fate of Progenitor Cells. Nat Commun (2019) 10:2549. doi: 10.1038/s41467-019-09992-3 31186409 PMC6560121

[30] Antonopoulos AS Tousoulis D. The Molecular Mechanisms of Obesity Paradox. Cardiovasc Res (2017) 113:1074–86. doi: 10.1093/cvr/cvx106 28549096

[31] Jung CH Lee WJ Song KH. Metabolically Healthy Obesity: A Friend or Foe? Korean J Intern Med (2017) 32:611–21. doi: 10.3904/kjim.2016.259 PMC5511946 28602062

[32] Alligier M Gabert L Meugnier E Lambert-Porcheron S Chanseaume E Pilleul F. Visceral Fat Accumulation During Lipid Overfeeding Is Related to Subcutaneous Adipose Tissue Characteristics in Healthy Men. J Clin Endocrinol Metab (2013) 98:802–10. doi: 10.1210/jc.2012-3289 23284008

[33] Yeoh AJ Pedley A Rosenquist KJ Hoffmann U Fox CS. The Association Between Subcutaneous Fat Density and the Propensity to Store Fat Viscerally. J Clin Endocrinol Metab (2015) 100:E1056-64. doi: 10.1210/jc.2014-4032 26062015 PMC4525002

[34] Atlantis E Martin SA Haren MT Taylor AW Wittert GA Members of the Florey Adelaide Male Ageing Study. Inverse Associations Between Muscle Mass, Strength, and the Metabolic Syndrome. Metabolism (2009) 58:1013–22. doi: 10.1016/j.metabol.2009.02.027 19394973

[35] Moon SS. Low Skeletal Muscle Mass Is Associated With Insulin Resistance, Diabetes, and Metabolic Syndrome in the Korean Population: The Korea National Health and Nutrition Examination Survey (KNHANES) 2009-2010. Endocr J (2014) 61:61–70. doi: 10.1507/endocrj.EJ13-0244 24088600

[36] Zisman A Peroni OD Abel ED Michael MD Mauvais-Jarvis F Lowell BB. Targeted Disruption of the Glucose Transporter 4 Selectively in Muscle Causes Insulin Resistance and Glucose Intolerance. Nat Med (2000) 6:924–8. doi: 10.1038/78693 10932232

[37] Frontera WR Ochala J. Skeletal Muscle: A Brief Review of Structure and Function. Calcif Tissue Int (2015) 96:183–95. doi: 10.1007/s00223-014-9915-y 25294644

[38] Primeau V Coderre L Karelis AD Brochu M Lavoie ME Messier V. Characterizing the Profile of Obese Patients Who Are Metabolically Healthy. Int J Obes (Lond) (2011) 35:971–81. doi: 10.1038/ijo.2010.216 20975726

[39] Velho S Paccaud F Waeber G Vollenweider P Marques-Vidal P. Metabolically Healthy Obesity: Different Prevalences Using Different Criteria. Eur J Clin Nutr (2010) 64:1043–51. doi: 10.1038/ejcn.2010.114 20628408

[40] van Vliet-Ostaptchouk JV Nuotio ML Slagter SN Doiron D Fischer K Foco L. The Prevalence of Metabolic Syndrome and Metabolically Healthy Obesity in Europe: A Collaborative Analysis of Ten Large Cohort Studies. BMC Endocr Disord (2014) 14:9. doi: 10.1186/1472-6823-14-9 24484869 PMC3923238

[41] Kramer CK Zinman B Retnakaran R. Are Metabolically Healthy Overweight and Obesity Benign Conditions?: A Systematic Review and Meta-Analysis. Ann Intern Med (2013) 159:758–69. doi: 10.7326/0003-4819-159-11-201312030-00008 24297192

[42] Arnlöv J Ingelsson E Sundström J Lind L. Impact of Body Mass Index and the Metabolic Syndrome on the Risk of Cardiovascular Disease and Death in Middle-Aged Men. Circulation (2010) 121:230–6. doi: 10.1161/CIRCULATIONAHA.109.887521 20038741

[43] Zhou Z Macpherson J Gray SR Gill JMR Welsh P Celis-Morales C. Are People With Metabolically Healthy Obesity Really Healthy? A Prospective Cohort Study of 381,363 UK Biobank Participants. Diabetologia (2021) 64:1963–72. doi: 10.1007/s00125-021-05484-6 PMC8382657 34109441

[44] Hwang YC Hayashi T Fujimoto WY Kahn SE Leonetti DL McNeely MJ. Visceral Abdominal Fat Accumulation Predicts the Conversion of Metabolically Healthy Obese Subjects to an Unhealthy Phenotype. Int J Obes (Lond) (2015) 39:1365–70. doi: 10.1038/ijo.2015.75 PMC4564328 25920773

[45] Hajian-Tilaki K Heidari B. Metabolically Healthy Obese and Unhealthy Normal Weight in Iranian Adult Population: Prevalence and the Associated Factors. Diabetes Metab Syndr (2018) 12:129–34. doi: 10.1016/j.dsx.2017.11.005 29196231

