# Supplementary material for: Association Between Indices of Body Composition and Metabolically Unhealthy Phenotype in China: A Cross-Sectional Study
Source: Front Endocrinol (Lausanne). 2022 May 9;13:891327. doi: 10.3389/fendo.2022.891327 (PMC9124857; doi:10.3389/fendo.2022.891327)
Supplement: Supplementary file 1 [file Table_1.docx]

**Supplemental table 1. Abdominal fat distribution and skeletal muscle in different metabolically phenotypes stratified by sex.**

|  | Normal weight |  |  | Overweight |  |  | Obese |  |  |
| --- | --- | --- | --- | --- | --- | --- | --- | --- | --- |
|  | Metabolically healthy | Metabolically  unhealthy | *P* value | Metabolically healthy | Metabolically  unhealthy | *P* value | Metabolically healthy | Metabolically  unhealthy | *P* value |
| **TAT** |  |  |  |  |  |  |  |  |  |
| Men | 162.44±67.71 | 179.66±64.07 | **0.001** | 255.78±63.47 | 288.38±61.35 | **＜0.001** | 349.31±74.81 | 369.85±76.67 | **0.012** |
| Women | 214.05±61.34 | 247.11±57.22 | **＜0.001** | 297.39±60.85 | 313.22±66.46 | **0.001** | 373.86±66.18 | 381.45±81.17 | 0.235 |
| Men vs. Women P value | ＜0.001 | ＜0.001 |  | ＜0.001 | ＜0.001 |  | 0.006 | 0.076 |  |
| **VAT** |  |  |  |  |  |  |  |  |  |
| Men | 84.95±35.00 | 95.65±37.94 | **0.001** | 125.71±37.78 | 150.21±42.11 | **＜0.001** | 154.34±51.21 | 181.05±52.68 | **＜0.001** |
| Women | 78.48±26.94 | 101.21±28.32 | **＜0.001** | 107.02±32.85 | 128.83±36.53 | **＜0.001** | 136.01±38.58 | 157.17±46.05 | **＜0.001** |
| Men vs. Women P value | 0.033 | 0.028 |  | ＜0.001 | ＜0.001 |  | 0.005 | ＜0.001 |  |
| **SAT** |  |  |  |  |  |  |  |  |  |
| Men | 77.49±38.77 | 84.02±33.73 | **0.019** | 130.07±39.25 | 138.17±35.75 | **0.012** | 194.97±57.44 | 188.80±46.22 | 0.935 |
| Women | 135.57±44.15 | 145.90±42.12 | **0.007** | 190.37±46.17 | 184.40±49.89 | 0.069 | 237.85±55.29 | 224.28±61.93 | **0.016** |
| Men vs. Women P value | ＜0.001 | ＜0.001 |  | ＜0.001 | ＜0.001 |  | ＜0.001 | ＜0.001 |  |
| **SMI** |  |  |  |  |  |  |  |  |  |
| Men | 97.67±16.26 | 96.47±14.43 | 0.275 | 96.56±16.13 | 94.77±16.04 | 0.121 | 103.96±22.85 | 103.68±16.58 | 0.403 |
| Women | 82.89±13.20 | 79.27±12.93 | **0.003** | 83.84±15.11 | 82.98±16.07 | 0.226 | 90.07±16.45 | 89.15±17.68 | 0.581 |
| Men vs. Women P value | ＜0.001 | ＜0.001 |  | ＜0.001 | ＜0.001 |  | ＜0.001 | ＜0.001 |  |

TAT: total adipose tissue; VAT: visceral adipose tissue; SAT: subcutaneous adipose tissue; SMI: skeletal muscle index.
